# Breeding Site Characteristics and Associated Factors of *Culex pipiens* Complex in Lhasa, Tibet, P. R. China

**DOI:** 10.3390/ijerph16081407

**Published:** 2019-04-18

**Authors:** Xiaobo Liu, Baimaciwang, Yujuan Yue, Haixia Wu, Pengcuociren, Yuhong Guo, Cirenwangla, Dongsheng Ren, Danzenggongga, Dazhen, Jun Yang, Zhaxisangmu, Jing Li, Cirendeji, Ning Zhao, Jimin Sun, Jing Li, Jun Wang, Cirendunzhu, Qiyong Liu

**Affiliations:** 1 State Key Laboratory of Infectious Disease Prevention and Control, Collaborative Innovation Center for Diagnosis and Treatment of Infectious Diseases, National Institute for Communicable Disease Control and Prevention, Chinese Center for Disease Control and Prevention, Beijing 102206, China; liuxiaobo@icdc.cn (X.L.); yueyujuan@icdc.cn (Y.Y.); wuhaixia@icdc.cn (H.W.); guoyuhong@icdc.cn (Y.G.); rendongsheng@icdc.cn (D.R.); zhaoning@icdc.cn (N.Z.); wangjun@icdc.cn (J.W.); 2 WHO Collaborating Centre for Vector Surveillance and Management, Beijing 102206, China; 3 Tibet Center for Disease Control and Prevention, Lhasa 850000, China; nc916@126.com (B.); pc911amcdc@163.com (P.); Kunten7755@163.com (D.); dazhen199478@163.com (D.); xzcdcywb@163.com (Z.); deji1011@126.com (C.); cirendunzhuok@126.com (C.); 4 Lhasa Chengguan District Center for Disease Control and Prevention, Lhasa 850000, China; cgqjkjd@163.com; 5 Institute for Environmental and Climate Research, Jinan University, Guangzhou 511443, China; yangjun_eci@jnu.edu.cn; 6 School of Public Health and Management, Weifang Medical University, Weifang 261053, China; lijingsddx@126.com; 7 Zhejiang Provincial Center for Disease Control and Prevention, Hangzhou 310051, China jmsun@cdc.zj.cn; 8 Changping District Center for Disease Control and Prevention, Beijing 102206, China; lijing986169323@sina.cn

**Keywords:** *Culex pipiens* complex, breeding characteristics, cross-sectional study, Tibet, mosquito control

## Abstract

Characterizing the breeding sites of *Culex pipiens* complex is of major importance for the control of West Nile disease and other related diseases. However, little information is available about the characteristics and associated factors of the breeding sites of the *Cx. pipiens* complex in Lhasa, a representative high-altitude region in Southwestern China. In this study, a cross-sectional study concerning the breeding site characteristics and associated factors of the *Cx. pipiens* complex was carried out in Lhasa, Tibet from 2013–2016. Chi-square analysis and binary logistic regression analysis were applied to identify the key factors associated with the presence of *Cx. pipiens* complex larvae. Using a standard dipping method, 184 water bodies were examined and *Cx. pipiens* complex larvae were observed in 36 (19.57%) of them. There were significant differences in the composition of *Cx. pipiens* complex larvae among the breeding site stability (χ^2^ = 19.08, *p* = 0.00) and presence or absence of predators (χ^2^ = 6.986, *p* = 0.008). Binary logistic regression analysis indicated that breeding site stability and presence or absence of predators were significantly associated with the presence of *Cx. pipiens* complex larvae in Chengguan District, Lhasa. Relatively permanent water bodies such as water bodies along river fringes, ponds and puddles, and water bodies with no predators should be paid more attention for future *Cx. pipiens* complex larvae abatement campaigns in Lhasa, China.

## 1. Introduction

*Culex pipiens* complex mosquitoes have a global distribution and are primary vectors of pathogens with public health significance including West Nile disease [1,2], St. Louis encephalitis [3], Sindbis [4], Rift Valley fever viruses [5,6], and periodic filariasis and encephalitis [7]. In China, the *Cx. pipiens* complex consists of four subspecies, including: *Cx. pipiens pipiens*, *Cx. pipiens quinquefasciatus*, *Cx. pipiens pallens,* and *Cx. pipiens molestus* [8].

Lhasa, the capital of the Tibet Autonomous Region (TAR) of China, is an international tourist city with plateau and national characteristics. It is known as one of the highest cities in the world, having an elevation of about 3650 meters, and lies in the center of the Tibetan Plateau. Evidence has shown that mosquitoes in the *Cx. pipiens* complex have already settled in urban Lhasa, TAR, and the local *Cx. pipiens* complex comprises the subspecies *Cx. pipiens pipiens*, *Cx. pipiens pallens*, *Cx. pipiens quinquefasciatus,* and hybrids of these subspecies. In addition, climate change may have played a role in the establishment of mosquitoes in Lhasa [9]. At present, the transmission of diseases by the *Cx. pipiens* complex has not been reported in Lhasa. However, further warming raises the risk of the outbreak of mosquito-borne diseases in the future [10,11]. This has already constituted a potential public health threat to the locals [12].

Each species of mosquito has its preferred breeding site for oviposition [13], depending on climate conditions, physical geography, and human activity [14]. Breeding sites can be natural or artificial, shaded or sunny, permanent or temporary, of various sizes, and found in running or stagnant water bodies, among others. Globally, many studies have displayed that the breeding habits of the *Cx. pipiens* complex are similar, and the main breeding sites are water bodies that are not seriously polluted, such as sinkholes, sewer ditches, cesspits with clear water, stagnant water in low-lying land, and so on [15]. The *Cx. pipiens* complex has better capacity of adaptation towards diverse breeding sites. In the Wroclaw area of Poland, evidence has shown that *Cx. pipiens* s.l. (L.) was well adapted to various breeding site types including ditches, catch basins, flowerpots, and buckets with diverse water quality [16].

Informed larval interventions that target more profuse breeding sites have enormous potential in combating *Cx.-pipiens-*complex-related diseases, especially at a regional scale. Though some studies have examined the types of *Culex* breeding sites and their related characterization in high-altitude regions [17], little information is available concerning the high altitude regions in China. This poses a serious challenge for the prevention and control of potential mosquito-borne diseases in the future. Therefore, this study aims to explore the breeding site characteristics of the *Cx. pipiens* complex and related environmental and physico-chemical parameters in urban Lhasa, to determine which breeding site characteristics can better explain the presence of *Cx. pipiens* complex larvae. The results of this study could provide first-hand scientific assessment of *Cx. pipiens* complex breeding sites and provide implications for developing intervention measures to control mosquito-borne diseases in Lhasa in the future.

## 2. Materials and Methods

### 2.1. Study Area

Lhasa City is an international tourist city with plateau and ethnic characteristics and is the administrative capital of the Tibet Autonomous Region of the People’s Republic of China, consisting of one municipal district (Chengguan District) and seven counties (Linzhou county, Dangxiong county, Nimu county, Qushui county, Duilongdeqing county, Dazi county, and Mozhugongka county).

**Figure 1:**
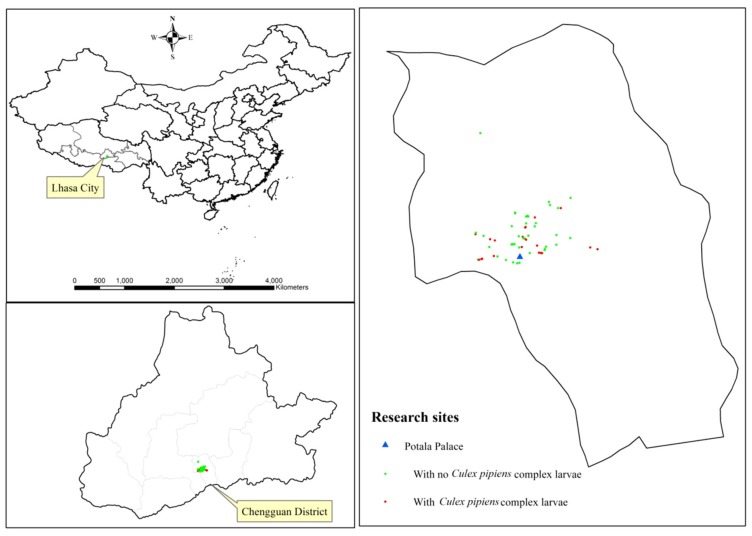
The research sites of this study.

This study was conducted in selected sites of Chengguan District, Lhasa from 2013–2016 (Figure 1). Chengguan District was the only municipal district in Lhasa city during the study period, with a population of 279,074 in 2013. By 2012, Chengguan District covered an area of 523 square km, but the municipal district only accounts for about 10% of the total area of Chengguan District. These sites were selected mainly according to the geographic and socio-economic characteristics of urban Lhasa. The selected research sites from 2013–2016 mentioned above are summarized in Table 1.

### 2.2. Mosquito Larvae Sampling and Identification

Based on our previous research [9], mosquito species of Chengguan District, Lhasa belong to the subspecies of the *Cx. pipiens* complex. Evidence has shown that the breeding sites of mosquito of the *Cx. pipiens* complex mainly include sinkholes, sewer ditches, cesspits, low-lying land, and so on, which generally exist in outdoor environments [15]. Therefore, the selection of potential breeding sites in this study mainly focused on outdoor surroundings.

The larval sampling was conducted using a standard dipping method [18]. In the outdoor surroundings, all the potential breeding sites were located and inspected. When mosquito larvae were present, 10 dips were taken with a dipper in each breeding site. When a breeding site was too small to make 10 dips, water was dipped as many times as possible. In large water bodies, dipping was carried out 100 m apart [14].

To further identify the species of the collected mosquito larvae in Lhasa, the late instars of mosquito larvae were immediately preserved in 90% absolute ethanol and then taken to the laboratory of the National Institute for Communicable Disease Control and Prevention (ICDC), the Chinese Center for Disease Control and Prevention (China CDC) [19]. A multiplex PCR protocol was adopted to identify the subspecies of mosquitoes using polymorphisms in the second intron of the acetylcholinesterase-2 (ace-2) locus, developed by Smith and Fonseca [20]. For the polymerase chain reaction (PCR) identification, the method used in this study was the same as in our previous research [9].

### 2.3. Breeding Site Characterization

Prior to the survey of potential breeding sites, information about the research sites was recorded, including geographic location, population, economic development level, water bodies, park, housing conditions, and land utilization. The larval breeding sites were characterized either visually or using hand-held equipment.

Some key breeding site characteristics, such as breeding site types, the location of water bodies, distance to the nearest household, perimeter of water body, breeding site stability, substrate types, predators, vegetation, nature (artificial or natural), water flow or static water, shade, water depth, pH, water temperature, dissolved oxygen, turbidity, soluble solids, conductivity, salinity, and resistance, were recorded or tested in this study.

Identified water bodies were classified according to their nature, classified as: river fringes (breeding sites formed along riverbanks when the water level drops), ponds (water area larger than 50 m^2^), puddles (water area less than 50 m^2^), irrigation or drainage ditches, and ground pools [21]. The perimeter of each breeding site was categorized by estimation as shorter than 1 m, 1–10 m, or longer than 10 m. Substrate types were classified into cement or concrete, soil, metal, and others. Distance to the nearest house was measured by GPS and classified as less than 10 m, 10–100 m, and greater than 100 m. Water depth was classified into greater than or equal to 0.5 m and less than 0.5 m. The stability of mosquito larval breeding sites was classified as either temporary or permanent. Temporary breeding sites held water for a short period of time (approximately two weeks after the rainy season ended) and stemmed mainly from rain showers. When rain ceased, these breeding sites dried out. On the other hand, the permanent breeding sites held water for a longer period of time (approximately two to three months after the rain ended or fed by natural underground sources) and hence were more stable.

pH, water temperature, dissolved oxygen, turbidity, soluble solids, conductivity, salinity, and resistance were recorded by handheld equipment. pH was recorded by a Waterproof pHTestr 30 (OAKTON Instruments, Vernon Hills, IL USA) [22]. Dissolved oxygen was recorded by portable dissolved oxygen meter (SG6-FK2 CN, Mettler-Toledo, LLC, Columbus, OH USA). To measure turbidity, turbidity meter was adopted in this study. Some indices, such as soluble solids, conductivity, salinity, and resistance were recorded by a portable multiparameter tester (SG23-FK-CN, Mettler-Toledo, LLC, Columbus, OH, USA).

Temperature (°C) and relative humidity (%) data were obtained from the China Weather Website (http://www.weather.com.cn). During collections, ambient outdoor air temperature and relative humidity were recorded by portable weather station (Davis Weather Link 6.0.3, Davis, CA, USA).

### 2.4. Ethics Statement

This study was approved by the Ethics Committee of China CDC (No. 201214). Ethical approvals were also obtained from the Lhasa Health Bureau, Chengguan District CDC and Tibet CDC respectively in the Tibet Autonomous Region.

### 2.5. Statistical Analysis

Chi-square test was applied to determine the importance of factors for explaining the presence or absence of *Cx. pipiens* complex larvae. Some factors with statistical significance in the chi-square analysis were selected to do further binary logistic regression analysis to calculate the odds ratio (OR) and 95% Wald confidence intervals. Presence of larvae was categorized as one, while the absence of larvae was categorized as zero in the logistic regression model. Statistical analysis was carried out using SPSS software (Version 19.0 for windows, SPSS Inc., Chicago, IL, USA). *p* < 0.05 was considered as statistically significant, and all tests were two-tailed.

## 3. Results

### 3.1. The Potential Mosquito Breeding Sites in Lhasa, 2013–2016

**Figure 2:**
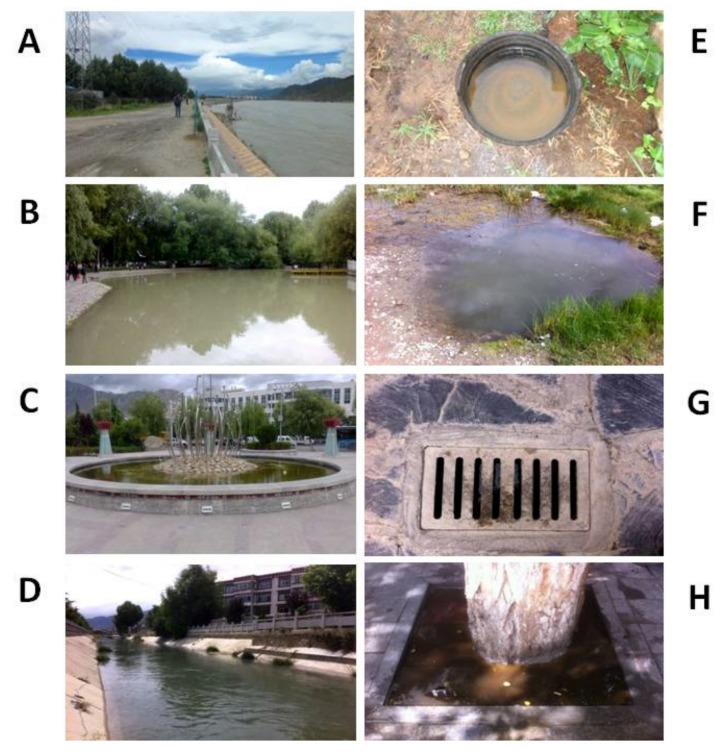
Representative water bodies in Lhasa City, Tibet, P. R. China. (**A**) River fringe; (**B**) Pond (≥50 m^2^); (**C**) Puddle (<50 m^2^); (**D**) Irrigation/drainage ditch; (**E**) Containers; (**F**) Temporary ground pool; (**G**) Sewer/tube well; (**H**) Other.

In this study, 184 potential mosquito breeding sites were examined from the sampled locations from 2013–2016. Representative water bodies in Lhasa are shown in Figure 2.

### 3.2. The Positive Breeding Sites and Species of Mosquito Larvae in Lhasa

Among potential mosquito breeding sites, *Cx. pipiens* complex larvae were observed in 37 water bodies, accounting for 20.1% of overall water bodies (Table 1). In total, 180 *Culex* larvae collected in 2013–2016 from 36 water bodies as mentioned above were further identified to subspecies according to a multiplex PCR identification. It was demonstrated that all the identified mosquito larvae belonged to subspecies of the *Cx. Pipiens* complex, including 63 pure mosquitoes (35%) and 117 hybrids (65%). The pure mosquitoes included 22 *Cx. Pipiens pipiens*, 11 *Cx. Pipiens quinquefasciatus,* and 30 *Cx. pipiens pallens*. Possible hybrids consisted of 80 *Cx. pipiens pipiens* × *Cx. pipiens pallens*, 26 *Cx. pipiens pallens* × *Cx. pipiens quinquefasciatus*, and 11 *Cx. pipiens pipiens* × *Cx. pipiens quinquefasciatus*. Sequence analysis confirmed the accuracy of multiplex PCR in this study.

### 3.3. The Main Characteristics of 184 Potential Mosquito Breeding Sites in Lhasa

Among 184 sites which contained water, 36 (19.57%) were productive for *Cx. pipiens* complex larvae, including 12 puddles, 6 sewer or tube wells, 5 ponds, 4 temporary ground pools, 3 river fringes, 3 irritation or drainage ditches, and 3 other water bodies. The frequency and percent composition of the main characteristics of 184 potential mosquito breeding sites in Lhasa, Tibet are shown in Table 2.

### 3.4. Positive Breeding Sites and Key Factors Associated with the Presence of Mosquito Larvae

There were significant differences in the composition of *Cx. pipiens* complex larvae among the breeding site stability (χ^2^ = 19.08, *p* = 0.00), and the presence or absence of predators (χ^2^ = 6.986, *p* = 0.008, Table 3). Based on the results of chi-square analysis, we found that no significant differences were observed in the presence of *Cx. pipiens* complex larvae among breeding site type, distance to the nearest house, artificial or natural, flow or static, perimeter, pH, dissolved oxygen, soluble solid, salinity, substrate type, presence or absence of predators, vegetation, shade, water depth, water temperature, turbidity, conductivity, or resistance.

### 3.5. The Findings of Binary Logistic Regression Analysis

To further exclude the confounding factors of the results of chi-square analysis, binary logistic regression analysis was adopted. Breeding site stability and the presence or absence of predators were found to be the key factors which determined the presence of mosquito larvae (Table 4).

## 4. Discussion

The precise identification of mosquito larvae species in Lhasa is of great importance not only for the study of the mosquito ecology but also for prevention and control of mosquito-borne diseases in future. In this study, a multiplex PCR method revealed that all identified mosquito larvae belonged to subspecies of the *Cx. pipiens* complex. This is consistent with the results from previous reports in Tibet [9].

This study found that the larvae of the *Cx. pipiens* complex mainly existed in river fringes, puddles, sewers, and temporary ground pools, although there were no significant differences of mosquito larvae among these water bodies. Except for the breeding sites along the river fringe, other water bodies were mainly artificial and represented major types of breeding sites in Lhasa. This indicated the possibly important infection locations of mosquito-borne diseases such as filariasis, West Nile disease, and other potential diseases in the future. The findings of this study are similar to the studies of Savage and Miller and Hribar in the USA [15,23,24]. Considering their findings, members of the *Cx. pipiens* complex readily breed in storm sewer catch basins, clean and polluted ground pools, ditches, animal waste lagoons, effluent from sewage treatment plants, and other sites that are slightly to very eutrophic or polluted with organic wastes. Generally, *Cx*. *pipiens quinquefasciatus* is associated with more eutrophic water than *Cx*. *pipiens*. Habitats along river fringes were more important during the dry season when the water levels reduced, and stagnant pools of water suitable for mosquito breeding were created [25].

In this study, among the 184 breeding sites which contained water, nearly one-fifth of water bodies were productive for mosquito larvae. Based on the literature review and our previous findings, the subspecies of the *Cx. pipiens* complex settled their populations in Lhasa in only a short time. Furthermore, evidence has shown that adult mosquito density in Lhasa has been low in recent years, with 1.47 mosquitoes per trap per hour in 2012 and 0.20 mosquitoes per trap per hour in 2013 (light traps), 3.06 mosquitoes per net per hour in 2012 (bed net traps), and 7.90 mosquitoes per person per hour in 2012 (labor hour method) [26]. Therefore, this may be the main reason for the low proportion of mosquito larvae in the examined water bodies during the study period.

Regarding the subspecies of the *Cx. pipiens* complex, we found that both pure Culex mosquitoes (35%) and their hybrids (65%) existed in the study sites of Lhasa city. These included pure Culex mosquitoes (*Cx. pipiens pipiens, Cx. pipiens quinquefasciatus*, and *Cx. pipiens pallens*) and their hybrids (*Cx. pipiens pipiens* × *Cx. pipiens pallens*, *Cx. pipiens pallens* × *Cx. pipiens quinquefasciatus*, and *Cx. pipiens pipiens* × *Cx. pipiens quinquefasciatus*). The findings mentioned above were identical to those in the previous research in this region [9,27].

In the present study, the stability of mosquito larval breeding sites was identified to be the key reason associated with the presence of *Cx. pipiens* complex larvae in Lhasa. We discovered that the majority of *Cx. pipiens* complex larvae were observed in permanent breeding sites, including along river fringes, ponds, and puddles. The results of the study are similar to the findings of two studies in western Kenya. Fillinger et al. found that semi-permanent and permanent habitats were suitable for the proliferation of Culicines and *Anopheles gambiae* sensu lato [28]. Fort Ternan et al. found that permanent habitats held water for a long period of time, and that after the rain these habitats were more preferred by the Culicines and Anopheline mosquitoes [25].

Predator presence/absence was another key factor associated with the presence of *Cx. pipiens* complex larvae. Predators of mosquito larvae may include larvivorous fish [29,30,31], tadpoles, frogs [32], water bugs, cyclopoids, dragonflies, and *Chironomus* larvae [33,34]. If they were found in breeding sites, mosquito larvae were generally absent [35,36]. Tranchida et al. found that native Argentinean cyclopoids (Crustacea: Copepoda) were predators of *Cx. pipiens* (Diptera: Culicidae) mosquitoes in La Plata, Argentina [37].

Other factors potentially affecting the presence of *Cx. pipiens* complex larvae included distance to the nearest house, artificial or natural, flow or static, perimeter, pH, dissolved oxygen, soluble solid, salinity, substrate types, vegetation, shade, water depth, water temperature, turbidity [38], conductivity, and resistance [39]. However, there were no marked differences in the presence of *Cx. pipiens* complex larvae among these variables mentioned above, and further study needs to be carried out in the future.

To date, many studies have found that some biological and physicochemical characteristics of larval habitats such as pH, water temperature, dissolved oxygen, turbidity, soluble solids, conductivity, salinity, and resistance were correlated with the presence of mosquito larvae [14,17,32,40,41,42]. One study in Egypt examined the effects of environmental parameters on larval population density [43], including pH, biological and chemical oxygen demands, daytime water temperature, plant growth, salinity, total organic matter, and concentrations of heavy metals. They found that *Cx. pipiens* larvae displayed high tolerance to elevated levels of heavy metals in sewage water and sewage or domestic waste. Besides, these breeding sites had compensatory effects, probably caused by their high nutrient levels. Muturi et al. found that *Cx. quinquefasciatus* was associated with turbid water in U.S.A. [44]. However, no significant association was detected between the presence of *Cx. pipiens* complex larvae and habitat related variables in this study. The potential reasons still need to be investigated in further studies.

This study mainly studied the potential breeding sites in outdoor environments, however, a small amount of discard containers in rooms and drain pits of tap water in the courtyard pose a potential threat under suitable conditions. Further study could also focus on the variations of the breeding habit of the *Cx. pipiens* complex along with the change of the ecological environment caused by the urbanization in Lhasa in recent years. Other factors such as heavy metallic elements and their compounds [45], orthophosphates, biochemical oxygen demand (BOD), radioactive substances, the contents of minerals and their compounds [46], and some microbial contents were not detected in the current study, and similar studies could focus on these.

Furthermore, we could not ignore the possible error from identifying subspecies of the *Cx. pipiens* complex using multiplex PCR method in itself. There has been some research undertaken using DNA barcoding [47] and protein profiling [48] to distinguish the subspecies of the complex mentioned above. Since the adopted primers in this study only focused on three forward primers (ACEquin, ACEpall, and ACEpip) and one backward primer (B1246s), plus the limitation of sampling to some extent, *Cx. pipiens molestus* was not detected as larvae in this study in Lhasa [49].

## 5. Conclusions

The present study found that breeding site stability and presence or absence of predators were two key influencing factors which were significantly related to the presence of *Cx. pipiens* complex larvae. Mosquito larvae of subspecies of the *Cx. pipiens* complex mainly bred in permanent water bodies, and the absence of predators may increase the probability of finding them. Therefore, permanent water bodies with no predators should be highly emphasized for future *Cx. pipiens* complex control campaigns in Lhasa.

## Figures and Tables

**Table 1:** Locations of positive larvae breeding sites in Lhasa, 2013–2016, Tibet.

Year of investigation	Research sites	Direction from the Potala Palace	No mosquito larvae	With mosquito larvae	Total
**2013**	Zongjiaolukang Park	North	4	0	4
	Tibet Medical College	North	4	0	4
	Gesanghua Park	North	2	0	2
	Lalu Wetland	Northwest	2	0	2
	Potala Palace Square	South	5	1	6
	Tibet TV Station	West	1	0	1
	Jinniu Park	West	1	0	1
	Lhasa River Fringe	South	1	3	4
	Road Close to Lhasa River	South	1	0	1
	Jiacuo Community	West	5	4	9
	Xiashasu Community	East	2	1	3
	Bakuo Subdistrict	East	2	0	2
	Tibet Tax Collection Bureau	North	0	1	1
	Jiaerxi, Niangre	Northwest	2	0	2
	**Subtotal**		**32**	**10**	**42**
**2014**	Lhasa River Fringe	South	6	0	6
	Fountain Close to Lhasa River	South	1	0	1
	Gesanghua Park	North	6	4	10
	Mingzhu Park	North	1	0	1
	First Aid Call Center, Tibet People’s Hospital	North	1	0	1
	Potala Palace Square	South	4	0	4
	120 First Aid, Tibet	North	3	2	5
	Zongjiaolukang Park	North	0	2	2
	**Subtotal**		**22**	**8**	**30**
**2015**	Gesanghua Park	North	1	1	2
	Zhaxi Community, Zhaxi Street	East	0	1	1
	Zaki Temple, Tibet	North	8	0	8
	Water Park, Sela North Road	North	3	0	3
	Shengcheng Park, Sela North Road	North	1	0	1
	Judicial Officer Hospital	North	1	0	1
	Lhasa City Environmental Protection Bureau North Suburb Transfer Station	North	1	0	1
	Lalu Community, Gongdelin Street	Northwest	1	0	1
	Lalu Wetland	Northwest	8	0	8
	Bakuo Neighborhood Committee	East	5	2	7
	Xiashasu Neighborhood Committee	East	7	1	8
	Danjielin Community, Bakuo Street	East	0	1	1
	Seladaguo Alley, Bakuo Street	East	3	0	3
	Langmamu, Bakuo Street	East	3	0	3
	Bakuo Community, Bakuo Street	East	0	1	1
	Raosai Community, Bakuo Street	East	1	1	2
	Bailin Community, Bakuo Street	East	0	1	1
	Lugu Community, Bakuo Street	East	4	0	4
	Lhasa River Fringe	South	2	0	2
	Yaowangshan Park	West	2	0	2
	Zongjiaolukang Park	North	4	0	4
	Yunzhong Park	Southwest	1	0	1
	Tongjian Community, Gamagongsang	East	4	0	4
	Najinglubei Community, Gamagongsang	East	1	0	1
	Luobulinka	West	3	0	3
	**Subtotal**		**64**	**9**	**73**
**2016**	Ancient Architectural Art Company	East	2	0	2
	The Lawn Outside, Luobulinka	west	4	1	5
	Construction Bank of China, Tibetan Branch	west	1	0	1
	Sichuan and Tibet Highway Monument	South	0	1	1
	Lhasa Riverside Park Artificial Lake	South	1	0	1
	Roadside of Lhasa Hotel	South	1	3	4
	Tibet Tax Collection Bureau	North	2	3	5
	Lalu Wetland	Northwest	5	0	5
	Gesanghua Park	North	0	2	2
	Ruyi Park	North	1	0	1
	Tianhai Household Goods Market	North	1	0	1
	Tibet Audit Office	North	1	0	1
	Mingzhu Park	North	1	0	1
	Tibet Medical College and Its Vicinity	North	3	0	3
	Zongjiaolukang Park	North	5	0	5
	Gold Cattle Park	West	1	0	1
	**Subtotal**		**29**	**10**	**39**
**Total**			**147**	**37**	**184**

**Table 2:** The frequency and percent composition of the main characteristics of 184 potential mosquito breeding sites in Lhasa, Tibet.

Variable ^1^	*N* ^2^	% ^3^	Variable ^1^	*N* ^2^	% ^3^
**Breeding site types**			**Breeding site stability**		
River fringe	8	4.3	Permanent (≥2 weeks)	104	56.5
Pond (≥50 m^2^)	38	20.7	Temporary (<2 weeks)	80	43.5
Puddle (<50 m^2^)	39	21.2	Substrate types		
Irrigation/drainage ditch	14	7.6	Cement/concrete	79	42.9
Container	7	3.8	Soil	79	42.9
Temporary ground pool	21	11.4	Metal	3	1.6
Footprint/tire mark	4	2.2	Ceramic	2	1.1
Sewer/tube well	37	20.1	Others	21	11.4
Others	16	8.7	**Presence or absence of predators**		
**Distance to the nearest house**			No	151	82.1
<10 m	61	33.2	Yes	33	17.9
10~100 m	86	46.7	**Vegetation**		
>100 m	37	20.1	No	133	72.3
**Artificial or natural**			Yes	51	27.7
Artificial	136	73.9			
Natural	48	26.1	**Shade**		
**Flow or static**			No	30	41.1
Flow	29	15.8	Yes	43	58.9
Static	155	84.2	**Water depth**		
**Perimeter**			≥0.5 m	86	46.7
<1 m	16	8.7	<0.5 m	98	53.3
1~10 m	80	43.5	**Water temperature (℃)**		
>10 m	88	47.8	<15.85	17	23.3
**pH (Quartiles)**			15.85~19.65	18	24.7
<7.0600	11	15.1	19.65~22.30	24	32.9
7.0600~7.2650	17	23.3	>22.30	14	19.2
7. 2650~7.3925	12	16.4	**Turbidity**		
>7.3925	33	45.2	<7.495	21	28.8
**Dissolved oxygen (mg/L)**			7.495~19.77	24	32.9
<5.71	25	34.2	19.77~95.9750	11	15.1
5.71~6.055	13	17.8	>95.9750	17	23.3
6.055~6.44	23	31.5	**Conductivity (µs/cm)**		
>6.44	12	16.4	<171.4	16	21.9
**Soluble solids (mg/L)**			171.40~198.6	11	15.1
<84.475	15	20.5	198.6~247.25	14	19.2
84.475~98.75	11	15.1	>247.25	32	43.8
98.75~121.775	13	17.8	**Resistance (Ω·cm)**		
>121.775	34	46.6	<3.535	30	41.1
**Salinity (ppt)**			3.535~5.025	19	26.0
<0.08	18	24.7	5.025~5.7625	11	15.1
0.08~0.10	15	20.5	>5.7625	13	17.8
0.10~0.135	11	15.1			
>0.135	29	39.7			

^1^ Potential mosquito breeding sites classified based on the main characteristics (breeding site types, the location of water bodies, distance to the nearest household, perimeter, breeding site stability, substrate types, presence or absence of predators, vegetation, artificial or natural, flow or static, shade, water depth, pH, water temperature, dissolved oxygen, turbidity, soluble solids, conductivity, salinity, and resistance); ^2^ *N* refers to the number of each characteristic; ^3^ % refers to the percent composition of the components.

**Table 3:** Positive parameters determining the presence of *Culex pipiens* complex larvae in water bodies in Lhasa by chi-square analysis.

Variable	No Larvae	With Larvae	χ^2^	*p*	Variable	No Larvae	With Larvae	χ^2^	*p*
**Breeding site types**					**Breeding site stability**				
River fringe	5	3	8.718	0.367	Permanent (≥2 weeks)	72	32	19.08	0.00
Pond (≥50 m^2^)	33	5			Temporary (<2 weeks)	76	4		
Puddle (<50 m^2^)	27	12			**Substrate types**				
Irrigation/drainage ditch	11	3			Cement/concrete	60	19		
Container	7	0			Soil	67	12		
Temporary ground pool	17	4			Metal	2	1		
Footprints/tire marks	4	0			Ceramic	2	0		
Sewer/tube well	31	6			Others	17	4		
Others	13	3			**Presence or absence of predators**				
**Distance to the nearest house**					No	116	32	6.986	0.008
<10 m	47	14	1.308	0.520	Yes	35	1		
10~100 m	69	17			**Vegetation**				
>100 m	32	5			No	109	39	0.705	0.401
**Artificial or natural**					Yes	24	12		
Artificial	108	28	0.347	0.556					
Natural	40	8			**Shade**				
**Flow or static**					No	23	32	0.048	0.526
Flow	24	5	0.118	0.731	Yes	7	11		
Static	124	31			**Water depth**				
**Perimeter**					≥0.5 m	71	15	0.463	0.496
<1 m	15	1	2.244	0.326	<0.5 m	77	21		
1~10 m	62	18			**Water temperature (℃)**				
>10 m	71	17			<15.85	11	6	1.905	0.592
**pH (Quartiles)**					15.85~19.65	14	4		
<7.0600	7	4	3.110	0.375	19.65~22.30	18	6		
7.0600~7.2650	12	5			>22.30	12	2		
7. 2650~7.3925	8	4			**Turbidity**				
>7.3925	28	5			<7.495	14	7	2.998	0.392
**Dissolved oxygen (mg/L)**					7.495~19.77	17	7		
<5.71	20	5	4.043	0.257	19.77~95.9750	10	1		
5.71~6.055	7	6			>95.9750	14	3		
6.055~6.44	18	5			**Conductivity (µs/cm)**				
>6.44	10	2			<171.4	11	5	4.906	0.179
**Soluble solids (mg/L)**					171.40~198.6	10	1		
<84.475	10	5	2.327	0.507	198.6~247.25	8	6		
84.475~98.75	10	1			>247.25	26	6		
98.75~121.775	9	4			**Resistance (Ω·cm)**				
>121.775	26	8			<3.535	22	8	0.359	0.949
**Salinity (ppt)**					3.535~5.025	14	5		
<0.08	13	5	5.273	0.153	5.025~5.7625	9	2		
0.08~0.10	14	1			>5.7625	10	3		
0.10~0.135	6	5							
>0.135	22	7							

**Table 4:** Positive parameters determining the presence of *Cx. pipiens* complex larvae in water bodies in Lhasa by logistic regression analysis.

Variables	B	S.E.	Walds	df	Sig	Exp(B)
Predator	−3.157	1.043	9.156	1	0.002	0.043
Breeding site stability	−2.652	0.566	21.991	1	0.000	0.070
Constant	2.373	0.700	11.497	1	0.001	10.728

B refers to regression coefficient; S.E. refers to Std.Error; Walds refers to Wald test; df refers to degree of freedom; Sig refers to significance; Exp(B) refers to the exponent of B.
